# Research on the Milling Characteristics of SBS Modified Asphalt Pavement with Different Service Years Using the Discrete Element Method

**DOI:** 10.3390/ma18143226

**Published:** 2025-07-08

**Authors:** Xiujun Li, Zhipeng Zhang, Hao Liu, Hao Feng, Heng Zhang, Fangzhi Shi, Zhi Gou

**Affiliations:** 1 School of Environment and Architecture, University of Shanghai for Science and Technology, Shanghai 200093, China; junzixiu@usst.edu.cn (X.L.); 13661513257@163.com (Z.Z.); 2235052109@st.usst.edu.cn (Z.G.); 2 China MCC5 Group Corp. Ltd., Chengdu 610063, China; 15283126360@163.com (H.L.); 15390119390@163.com (H.Z.); 3 Road Engineering Technology Research Institute Co., Ltd., Jiaxing 314000, China; alan.shi@wirtgen-group.com

**Keywords:** SBS modified asphalt pavement, discrete element method, milling simulation, particle bonding ratio, milling rotor average force

## Abstract

The service years of the milled pavement are varied in numerous SBS modified asphalt pavement milling assignments. To investigate the milling characteristics of SBS (styrene–butadiene–styrene) modified asphalt pavements with different service years, the values of the bonding parameters were calibrated and verified and then used to build three simulation models for the milling of old asphalt pavements with service years of 2~3 years, 7~8 years, and 11~12 years, respectively. The milling characteristics of SBS modified asphalt pavements with different service years were investigated using the moving speed v and rotating speed ω of the milling rotor as test factors, and the particle bonding ratio ($R_{b}$) and rotor average force ($F_{a}$) as test indexes. The results demonstrate that the following: The regularity of the effects of milling rotor moving speed and rotating speed on the particle bonding ratio and milling rotor average forces remained consistent overall as the pavement age increased. For the same milling parameters, the particle bonding ratio and the rotor average force are reduced. From 2~3 years old pavements to 7~8 years old pavements, the overall reduction in the particle bonding ratio indicator is about 12%, and the average force on the milling rotor is about 24%. From 7~8 years old pavements to 11~12 years old pavements, the overall reduction in the particle bonding ratio indicator is about 3%, and the average force on the milling rotor is about 15%.

## 1. Introduction

**Figure 1:**
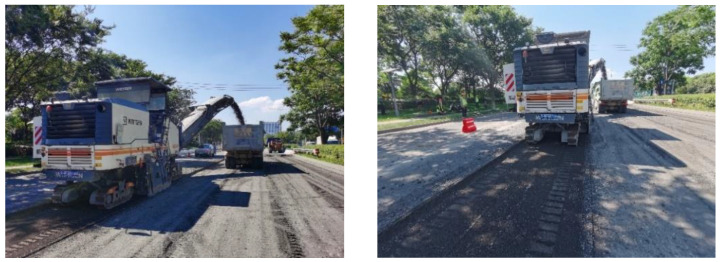
Asphalt pavement milling operation.

SBS modified asphalt mixtures are widely used in high-grade pavements due to the excellent high-temperature stability, low-temperature crack resistance, and fatigue resistance [1,2]. With the growth of SBS modified asphalt pavement service years, its road performance is gradually degraded, as not only does the overall appearance of the pavement have a certain impact, but also the SBS modified asphalt pavement service life and traffic safety pose a serious threat [3]. To effectively maintain the SBS modified asphalt pavement to be smooth and clean and extend its service life and ensure traffic safety, SBS modified asphalt pavement needs to be repaired and maintained. Milling operations are an important part of SBS modified asphalt pavement repair and maintenance work (Figure 1) and are primarily used to remove the aged and damaged layers from the road surface, providing the basis for subsequent renovation and rehabilitation work [4].

A large quantity of reclaimed asphalt pavement (RAP) is produced during milling operations. Efficient recycling of RAP not only reduces the waste of resources and protects the environment, but also creates considerable economic benefits, which is an inevitable choice for sustainable development of the transportation industry [5]. However, during milling works on SBS modified asphalt pavements with different service years, research on the agglomeration rate of recycled materials and the force exerted on the rotor is still unclear at this stage. The selected milling parameters (milling rotor moving speed, milling rotor rotating speed, etc.) are often the same or based on the subjective experience of the worker. Inadequate adaptation of milling rotor parameters to pavement conditions causes adverse consequences: (1) high-hardness coarse aggregate breaks during milling, increasing mechanical wear and wasting stone resources; (2) the produced RAP develops a high agglomeration rate, drastically lowering its recycling rate. Therefore, there is a need to study the milling characteristics of asphalt pavements with different service years.

Related international literature studies include the following: K. Diouri et al. [6] used viscoelastic and Drucker–Prager models to simulate milling of hot mix asphalt mixtures with different aging times at two temperatures, 25 °C and 60 °C, and showed that the stress zones generated by milling increased significantly with the increase in stiffness/aging. M. Zaumanis et al. [7] studied the effect of milling on asphalt pavements by varying three parameters of the milling machine (moving speed, milling depth, and rotating speed) at the construction site and found that an increase in the moving speed of the milling machine produces a larger agglomerated RAP at a greater milling depth. C. Song et al. [8] constructed a simulation model of old asphalt pavement using the discrete element method (DEM) to investigate the whole milling rotor. The effects of milling parameters on particle size were explored, and the results showed that the appropriate milling moving speed and rotating speed could effectively regulate the particle size. Y. Yao et al. [9] found through discrete element method (DEM) simulation and mechanical analysis that aggregate crushing occurs mainly in the trajectory of the cutter tip and during crack expansion, and agglomeration is mainly related to the area of the sliding surface. The milling speed and depth positively affect the agglomeration of RAP and on the contrary negatively affect the aggregate crushing; the milling rotating speed has little effect on these phenomena. M. G. Petrescu et al. [10] compared the asphalt pavement milling simulation results with indoor experimental results using the discrete element method (DEM) with varying milling depths, rotating speeds, and moving speeds, and found that the DEM simulation results were inconsistent with the experimental results; the optimal combination of milling parameters was determined.

The researchers have already achieved fruitful results after unremitting efforts, laying a solid foundation for asphalt pavement milling research. The milling simulation based on the discrete element method not only saves the test cost but also greatly improves the research efficiency. In a lot of asphalt pavement repair and maintaining projects, the service years of asphalt pavements milled by milling machines are different, but as far as the existing research results are concerned, none of them consider the influence of the aging degree of the pavement on the milling characteristics, which will lead to a mismatch between the results of the simulation and the actual engineering situation. This research adopts the discrete element simulation software EDEM 2023 to establish milling simulation models for SBS modified asphalt pavements with different service years to investigate the milling characteristics, aiming to provide a theoretical basis for milling operations of asphalt pavements with different service years and then improve the recycling utilization of RAP. This is essential for the sustainable development of the road industry. At the same time, this research reveals the mechanism of SBS modified asphalt pavement milling simulation and provides high-density experimental data, which provides explainable optimization goals, training data basis, and algorithm design basis for artificial intelligence-driven milling parameter adjustment. Artificial intelligence technology converts this information into dynamic strategies to achieve a “perception–decision–control” closed loop, significantly improving processing efficiency and quality in complex working conditions.

## 2. Methodology

**Figure 2:**
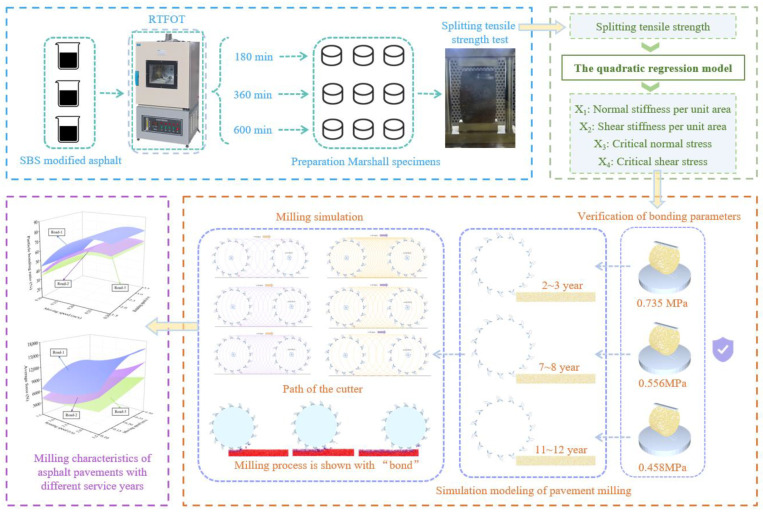
Technical line of research.

The technical route of this research is shown in Figure 2. Firstly, Marshall specimens were prepared by aged SBS modified asphalt with different RTFOT (Rolling Thin Film Oven Test) time lengths; then, an indoor splitting tensile strength test was conducted and the values of splitting tensile strength were substituted into the quadratic regression prediction equations to obtain the values of the four bonding parameters (normal stiffness per unit area, tangential stiffness per unit area, critical normal stress, and critical tangential stress) in the Hertz–Mindlin with bonding model. Following that, a splitting tensile strength test model was established in discrete element simulation software EDEM 2023 to verify the values of the bonding parameters, and the verified values of the bonding parameters were used to establish the EDEM simulation model of the SBS modified asphalt pavements with different service years. Finally, the milling characteristics of SBS modified asphalt pavements with different service years were investigated using the moving speed v and rotational angular speed ω of the milling rotor as the test factors, and the particle bonding ratio ($R_{b}$) and the rotor average force ($F_{a}$) as the test indexes.

### 2.1. Calibration of Bonding Parameters for EDEM Simulation Models

#### 2.1.1. Rolling Thin Film Oven Test

The SBS modified asphalt selected for this research was provided by a company in Jiaxing, China, according to the JTG E20-2011 [11] standard requirements of its three major technical indicators for testing; the test results are shown in Table 1.

**Figure 3:**
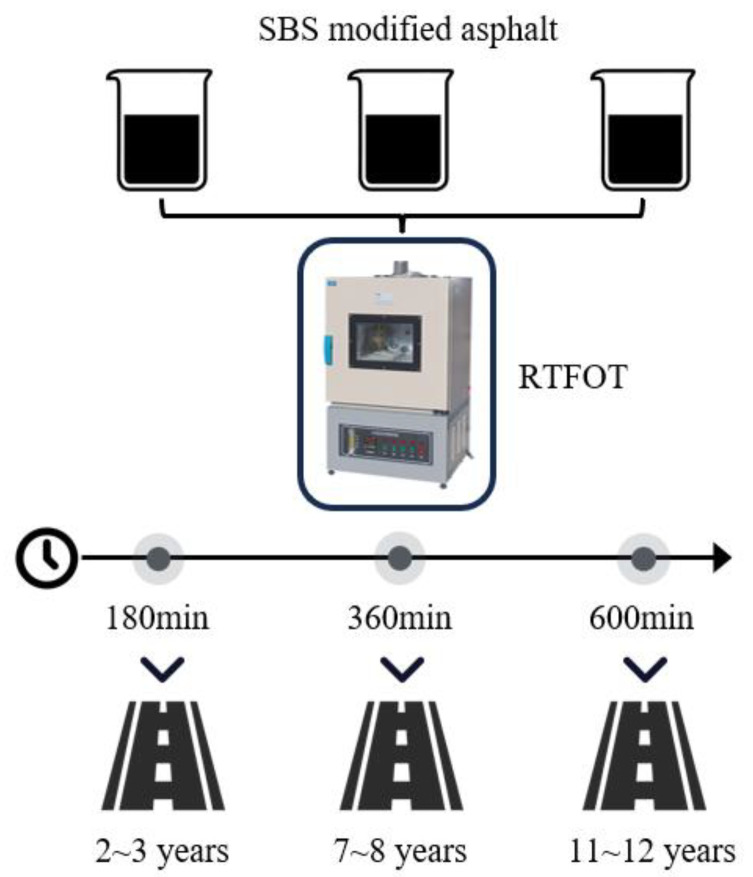
Relationship between SBS modified asphalt RTFOT aging time and actual service years of asphalt pavement.

The natural aging process of asphalt is extremely slow, so researchers usually use different indoor aging methods to simulate and accelerate the aging process of asphalt in related studies [12]. RTFOT, as a common method for asphalt aging simulation, has high test accuracy and good reproducibility and can better reflect the properties of asphalt after aging [13]. Zhang et al. [14] concluded from a comparative analysis that there is little difference in the rheological and microscopic properties of SBS modified asphalt under natural aging and RTFOT aging. Li [15], after comprehensive consideration of the indicators, concluded that the asphalt aging effect of RTFOT aging of 180 min is comparable to the aging degree of the pavement with an actual service life of 2 to 3 years, and that the asphalt aging effect of RTFOT aging of 270 min is similar to the aging degree of the pavement with a service life of 6 years. Lu [16] found that the degree of deterioration of RTFOT under 180 min conditions was comparable to the deterioration of pavement asphalt after 2 to 3 years of actual service, while the degree of deterioration of RTFOT under 270 min conditions coincided with the deterioration of pavement asphalt after 5 to 6 years of service. The findings of the two researchers corroborate each other. Li and Chen [17,18] investigated the viscoelasticity and deformation capacity of asphalt mastic after aging as well as the mastic–aggregate interfacial adhesion and compared it with the asphalt performance of actual pavements. It was concluded that the performance of asphalt mastic after RTFOT aging for 360 min is similar to the aging of actual asphalt pavements in service for 7 to 8 years. The pavement milling mechanism corresponding to a service life of 6 years was analyzed in terms of adhesion of the mastic. Yao et al. [19] found that the SBS modified asphalt indexes obtained from RTFOT aging of 4.8 h were equivalent to pavement asphalt with a service life of 5 to 6 years, while RTFOT aging of 5 h was consistent with SBS modified asphalt with a service life of 7 years. Wu et al. [20] adopted the RTFOT aging of SBS modified asphalt (equivalent to an asphalt pavement with a service life of about 11 to 12 years) for 600 min to produce aging asphalt mixture specimens and conducted indoor uniaxial compression tests and discrete element simulation tests to determine the parameters of the discrete element simulation model. The milling parameters were investigated after building a simulation model of the old asphalt pavement with the values of this parameter. In summary, the relationship between the SBS modified asphalt RTFOT aging time and the actual service years of asphalt pavement is summarized in Figure 3.

The asphalt rotating film oven equipment was used to simulate the indoor aging of SBS modified asphalt based on correspondence according to the RTFOT test method in the JTG E20-2011 [11]. This section provides a clearer distinction between the milling characteristics of SBS modified asphalt pavements with different service years. The aging times were 180 min, 360 min, and 600 min, respectively, which corresponded to the pavement’s actual service life of 2 to 3 years, 7 to 8 years, and 11 to 12 years, in turn. The aged SBS modified asphalt three major indicators were tested, and the test results are shown in Table 2.

#### 2.1.2. Indoor Splitting Tensile Strength Test

According to JTG 3432-2024 [21], the technical indexes of coarse aggregate, fine aggregate, and mineral powder were tested, and the test results are shown in Table 3. All the indexes meet the requirements of relevant technical standards.

**Figure 4:**
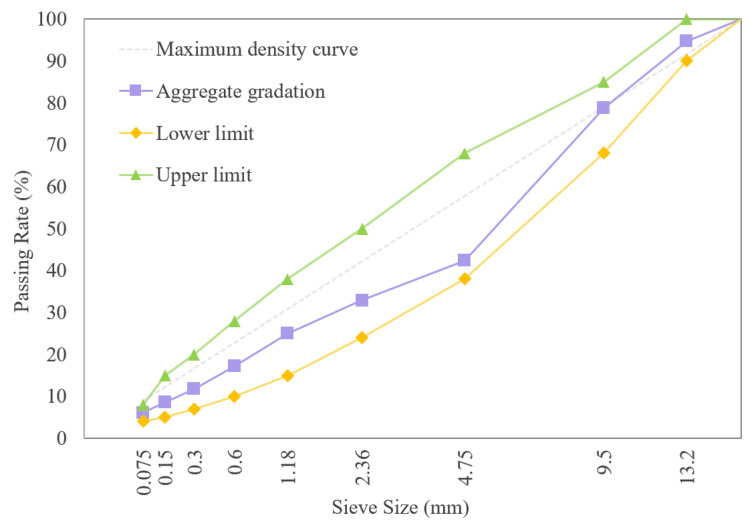
Gradation curve of SBS modified asphalt mixture.

The SBS modified asphalt mixture gradation in this research is AC-13 as an example, with an oil/stone ratio of 5%. The grading curves are shown in Figure 4. Marshall specimens with sizes of ϕ 101.6 mm ± 0.25 mm × 63.5 mm ± 1.3 mm were formed by the standard compaction method using SBS modified asphalt with different RTFOT aging times for splitting tensile strength tests.

The splitting tensile strength test of Marshall specimens was tested using the SYD-0730A multifunctional automatic asphalt pressure tester (Produced by Shanghai Changji Geological Instrument Co., Ltd, Shanghai, China) at a temperature of 15 °C and a loading rate of 50 mm/min. The splitting tensile strength is calculated according to Equation (1) from the JTG E20-2011 [11]. Three samples were tested in each test. The results of the calculations are shown in Table 4.

(1) $$R_{T}=0.006287P_{T}/h$$

where $R_{T}$ is the splitting tensile strength, MPa; $P_{T}$ is the maximum value of specimen load, N; and h is the height of specimen, mm.

#### 2.1.3. Calibration and Verification of Bonding Parameters

**Figure 5:**
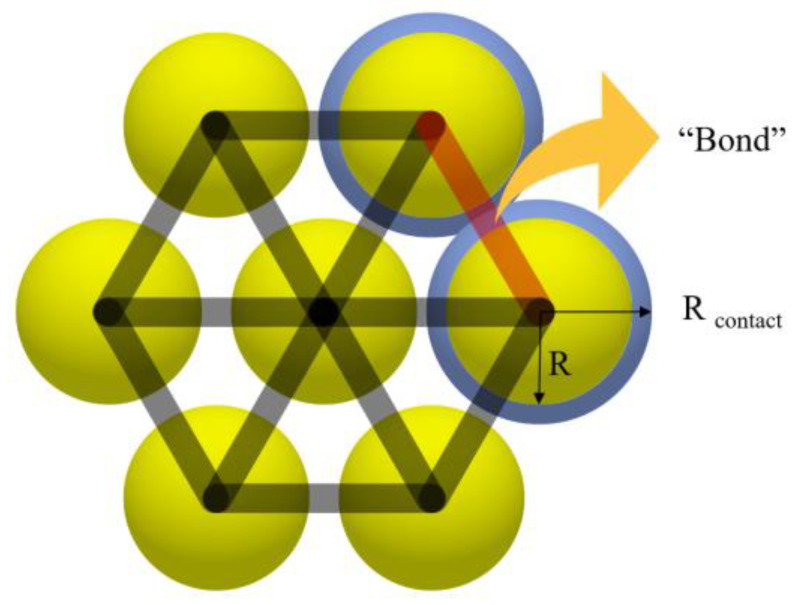
A schematic diagram of the “Bond”.

The key to constructing a simulation model for asphalt mixtures is to accurately model the bonding between particles [22]. The Hertz–Mindlin with bonding contact model incorporated in the discrete element simulation software EDEM 2023 is based on the Hertz–Mindlin model. The concept of “Bond” was introduced to simulate the bonding between particles. Although the chemical composition of asphalt as a binder cannot be reflected [23], bonding models are particularly useful in simulating fracture damage in asphalt concrete and geotechnical structures [24,25]. As shown in Figure 5, R is the physical radius of the particles and $R_{contact\;}$ is the contact radius of the particles. The “Bond” is generated when the distance between particles is less than the sum of the contact radiuses, which corresponds to a finite-size binder that resists a certain degree of normal and tangential motion. The “Bond” is broken when the critical values of normal and tangential stresses are exceeded and will not be generated again.

The calibration of four key bonding parameters of the Hertz–Mindlin with bonding model is required to establish a logical and reliable EDEM simulation model for asphalt mixtures. Li et al. [26] determined the quadratic regression equation of splitting tensile strength of asphalt mixtures with four binding parameters, namely normal stiffness per unit area ($X_{1}$), tangential stiffness per unit area ($X_{2}$), critical normal stress ($X_{3}$), and critical tangential stress ($X_{4}$), by response of the surface methodology. The quadratic regression model is

$$\begin{array}{c} R_{T}=-0.1486+\left(1.14\times 10^{-11}\right)X_{1}+\left(2.64\times 10^{-11}\right)X_{2}+\left(8.44\times 10^{-11}\right)X_{3}\\ +\left(5.41\times 10^{-11}\right)X_{4}-\left(5.22\times 10^{-22}\right)X_{1}X_{2}\\ -\left(2.95\times 10^{-21}\right)X_{1}X_{3}-\left(2.59\times 10^{-21}\right)X_{1}X_{4}\\ +\left(3.04\times 10^{-21}\right)X_{2}X_{3}+\left(6.11\times 10^{-21}\right)X_{3}X_{4}\\ -\left(7.01\times 10^{-22}\right)X_{2}^{2}-\left(5.68\times 10^{-21}\right)X_{4}^{2}\; \end{array}$$

The values of the four bonding parameters can be obtained by substituting the splitting tensile strength of the Marshall specimens in Table 4 into the quadratic regression equation, as shown in Table 5.

**Figure 6:**
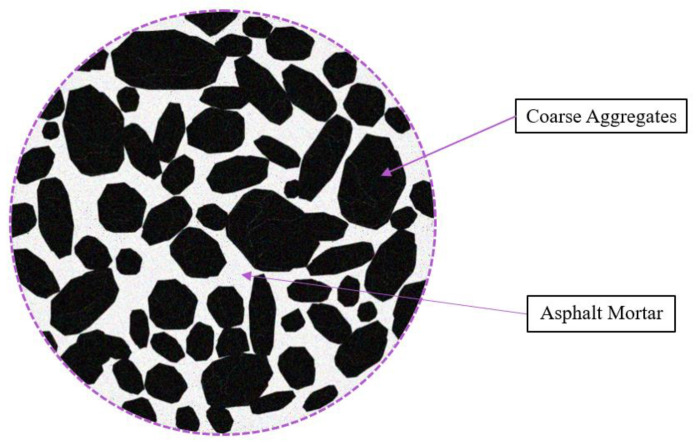
Schematic diagram of asphalt mixture mastic theory (coarse dispersion system).

The EDEM simulation model for the splitting tensile strength test was established in this research to verify the accuracy of the values of the bonding parameters. Asphalt mixtures are divided into coarse aggregate and asphalt mortar based on the asphalt mixture mastic theory (coarse dispersion system), as shown in Figure 6. The asphalt mortar consists of fine aggregate, filler, and asphalt [27].

**Figure 7:**
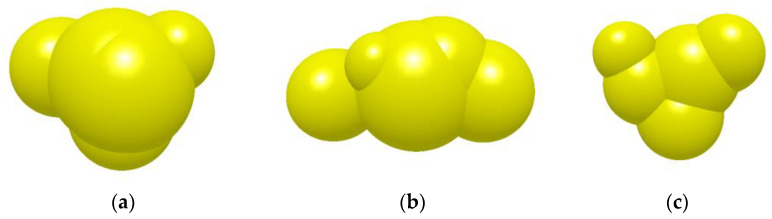
Discrete element model of three typical particles of coarse aggregate: (**a**) regular particle; (**b**) long particle; (**c**) flat particle.

The coarse aggregate is represented by three types of typical particles: regular particles, long particles, and flat particles [28]. The researchers found that as the number of filled spheres increased, the particle model got closer to the actual shape, but the computational accuracy did not change significantly [29]. The five-spherical particle model was chosen to simulate the real coarse aggregate particles to ensure the calculation accuracy and efficiency at the same time, as shown in Figure 7. The particle density of coarse aggregate is 2600 kg/m^3^. Asphalt mortar is represented by single-ball particles, with a density of 2400 kg/m^3^.

**Figure 8:**
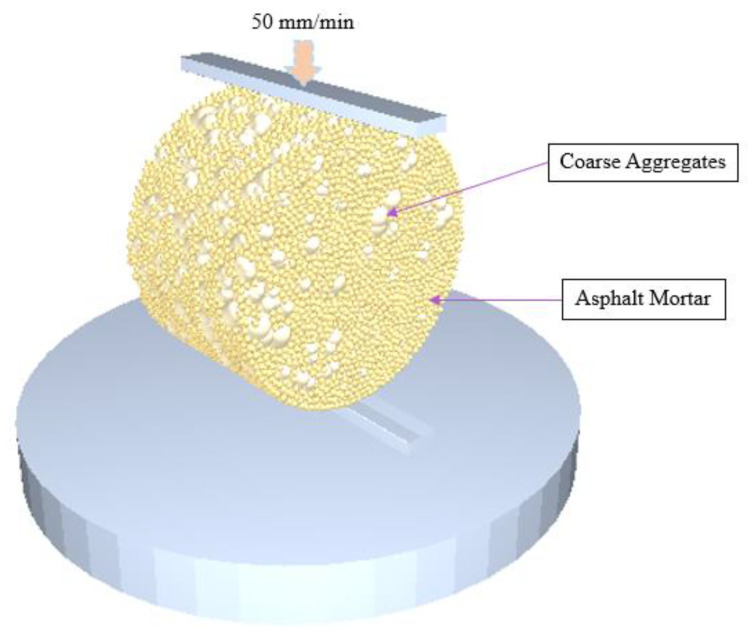
The EDEM simulation model of the splitting tensile strength test.

The particles are generated and compacted after setting the particle plant according to the SBS modified asphalt mixture gradation. The press bar model was imported and the loading rate was set to 50 mm/min. The EDEM simulation model for the splitting tensile strength test is shown in Figure 8.

The splitting tensile strength EDEM simulation tests were conducted sequentially after setting the bonding parameters according to Table 5. The simulation time for each scenario is 6 s, and no repeated simulations were performed. The splitting tensile strengths of 0.735 MPa, 0.556 MPa, and 0.458 MPa were calculated by using the post-processing module for each value of bonding parameters, respectively. The relative errors to the indoor test results were 3.54%, 4.81%, and 2.97%, respectively. The relative errors are small, proving the reliability of the values of the bonding parameters and the DEM models.

### 2.2. Research on Milling Characteristics of Old Asphalt Pavement

**Figure 9:**
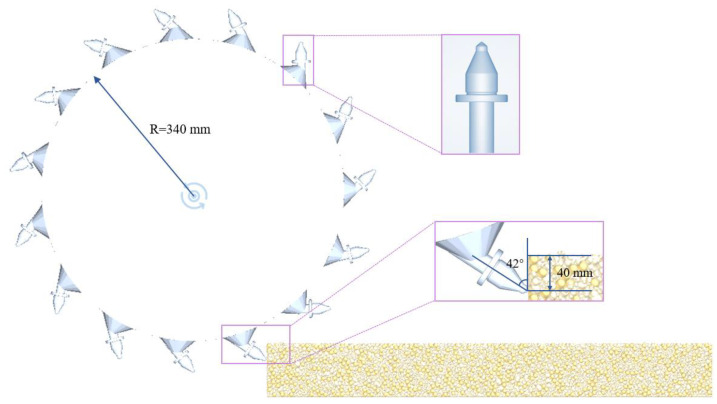
The EDEM simulation model for pavement milling.

The EDEM simulation model with dimensions of 1000 mm × 100 mm × 150 mm was generated using the EDEM 2023 simulation software according to the gradation of SBS modified asphalt mixtures, considering the boundary effects and time cost. The number of particles contained in the model is 24,082 and the number of “Bond” is 145,205. The previous research experience pointed out that the cutter tip at a 42° cutting angle has less wear and more stable operation [20,30,31]. With reference to Song [8] and Yao [9] as well, a cutting angle of 42° and milling depth of 40 mm were chosen for this research. The milling rotor and cutter tip were modeled by SolidWorks 2018 software and saved as stl. file format and then imported into the pavement model. The EDEM simulation model for pavement milling is shown in Figure 9.

The three sets of bonding parameter values of the pavement model were set and named Road-1, Road-2, and Road-3 according to Table 5, representing old asphalt pavements with 2~3, 7~8, and 11~12 years of service life, in turn. The movement of the cutter tip in the road milling process includes moving and rotating, which are controlled by the moving speed v and rotating speed ω of the milling rotor, respectively. The two are very important milling parameters, which not only have a greater impact on the wear of the cutter tip but also have a significant impact on the agglomeration rate of RAP. According to relevant research experiences [7,8], the milling rotor moving speeds used in this research were 0.1 m/s, 0.2 m/s, and 0.3 m/s; and the rotating speeds were 1.5 r/s, 2.5 r/s, and 3.5 r/s. With the moving speed and rotating speed of the milling rotor as the test factors and the particle bonding ratio and the rotor average force as the test indexes to set up the test program, the milling simulation research was carried out on three old asphalt pavement models in turn according to the test program, which is shown in Table 6.

## 3. Results and Discussion

**Figure 10:**
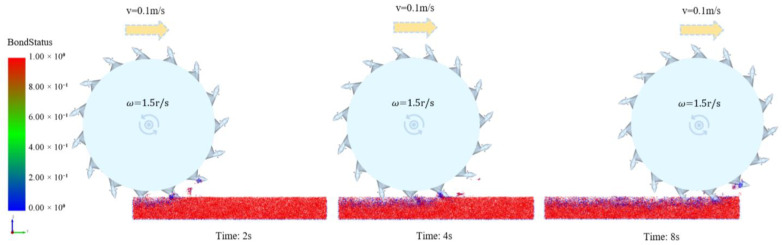
The milling process according to Bond Status.

The discrete element simulation software EDEM 2023 was used to simulate the three pavement models sequentially according to the test program. In the first group of simulation tests, for example, set the hidden particles and display the milling process with the Bond Status, as shown in Figure 10. Red indicates that the “Bond” is not under stress, while blue indicates that it has broken. Figure 10 clearly shows that after the milling rotor has been operated, the “Bond” is broken by the milling rotor; the milled particles fly out at a certain speed along the tangent direction of the milling rotor and some of the particles are still bonded together because the “Bond” is not broken, forming agglomerated RAP. As time passed, the number of breaks in “Bond” increased.

**Figure 11:**
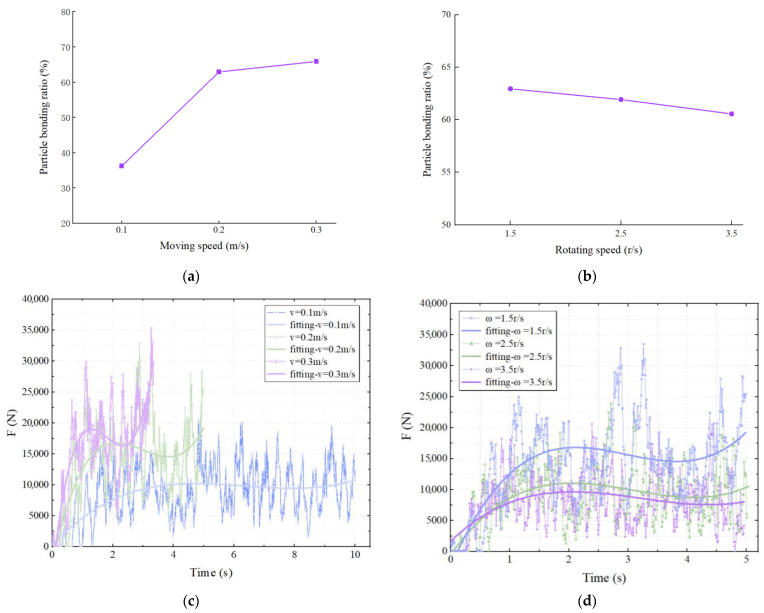
Effect of milling rotor parameters on two test indexes: (**a**) effect of moving speed on particle bonding ratio (ω= 1.5 r/s); (**b**) effect of rotating speed on particle bonding ratio (v = 0.2 m/s); (**c**) effect of moving speed on rotor forces (ω = 1.5 r/s); (**d**) effect of rotating speed on rotor forces (v = 0.2 m/s).

After the milling simulation was completed, the test data were exported by the post-processing module of the EDEM 2023 and calculated to obtain the particle bonding ratio and the rotor average force of the milling rotor. The general consistency of the milling moving speed and rotating speed on the particle bonding ratio and rotor average force was found when analyzing their effects under the three pavement model conditions. Therefore, using the Road-2 pavement model as an example, the effects of the milling rotor parameters on the two test indexes are shown in Figure 11.

From Figure 11, it can be clearly seen that when the milling rotor rotating speed is fixed, the particle bonding ratio and the milling rotor force are both increasing with the milling rotor moving speed, and at the same time, the fluctuation amplitude of the milling rotor force will increase accordingly; it is worth noting that the growth amplitude is reduced. The milling rotor moving speed is controlled to be constant, and as the milling rotor rotating speed increases, the particle bonding ratio and the milling rotor force situation will show a certain degree of decrease, but the decrease is limited.

**Figure 12:**
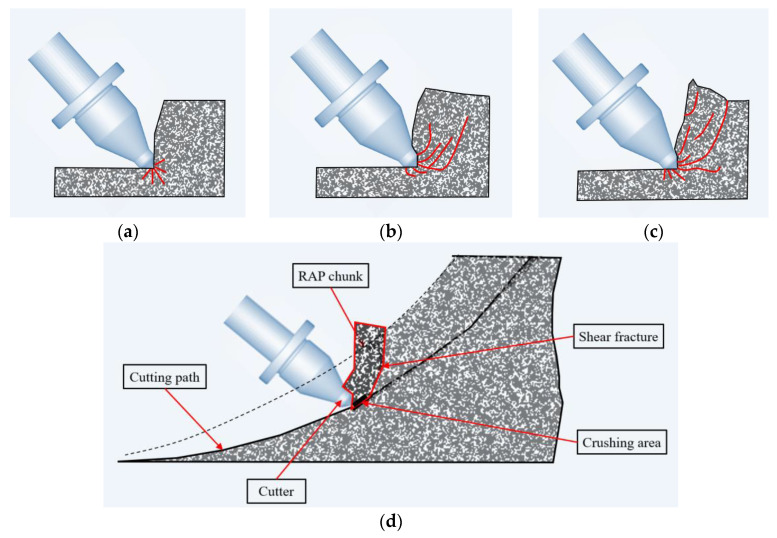
Schematic diagram of asphalt mixture milling process [32]: (**a**) impact and extrusion deformation stage; (**b**) shear fracture expansion stage; (**c**) asphalt mixture collapse stage; (**d**) milling process for asphalt mixtures.

Wang [32] pointed out that the process of the high-speed impact milling asphalt mixture is extremely short: the cutter tip contact with the asphalt mixture to its collapse can be divided into the impact and extrusion deformation, shear fracture expansion, and asphalt mixture collapse of the three stages, as shown in Figure 12a–c. (a) Impact and extrusion deformation stage: The tip of the cutter is wedged into the asphalt mixture at a certain speed, then the tip of the cutter in contact with the mixture under high positive pressure elasticity, plastic deformation, and aggregate break. The tip of the cutter can be wedged into the asphalt mixture so that the cutter tip contact with the asphalt mixture produces cracks. (b) Shear fracture expansion stage: The front surface of the cutter tip and the side of the asphalt mixture is the shearing stress state, where the stress pattern is close to the direct shear stress pattern, and the front surface of the cutter tip and the side of the asphalt mixture come in contact with the compressive stress under the action of the shear fracture and fracture expansion. As the asphalt binding material bond’s destructive force is small and mineral aggregate crushing requires more energy, according to the principle of minimum energy, an asphalt mixture main fracture is formed along the aggregate bonding surface fracture expansion. (c) Asphalt mixture collapse stage: Concerning the main fracture along the bonding surface under the continuous cutter tip expansion to the free surface, the front and side of the cutter tip has produced a fracture asphalt mixture in the free space above the slip and collapsed at a certain speed, resulting in crushing dust discharge. The front surface of the cutter tip and the side of the contact with the asphalt mixture continues to produce new shear fracture and fracture expansion. The formation of RAP agglomerates originated from the decomposition of the milling path surface, as shown in Figure 12d, and RAP agglomerates are more likely to form through fracture expansion when the surface area of the milling path is large.

**Figure 13:**
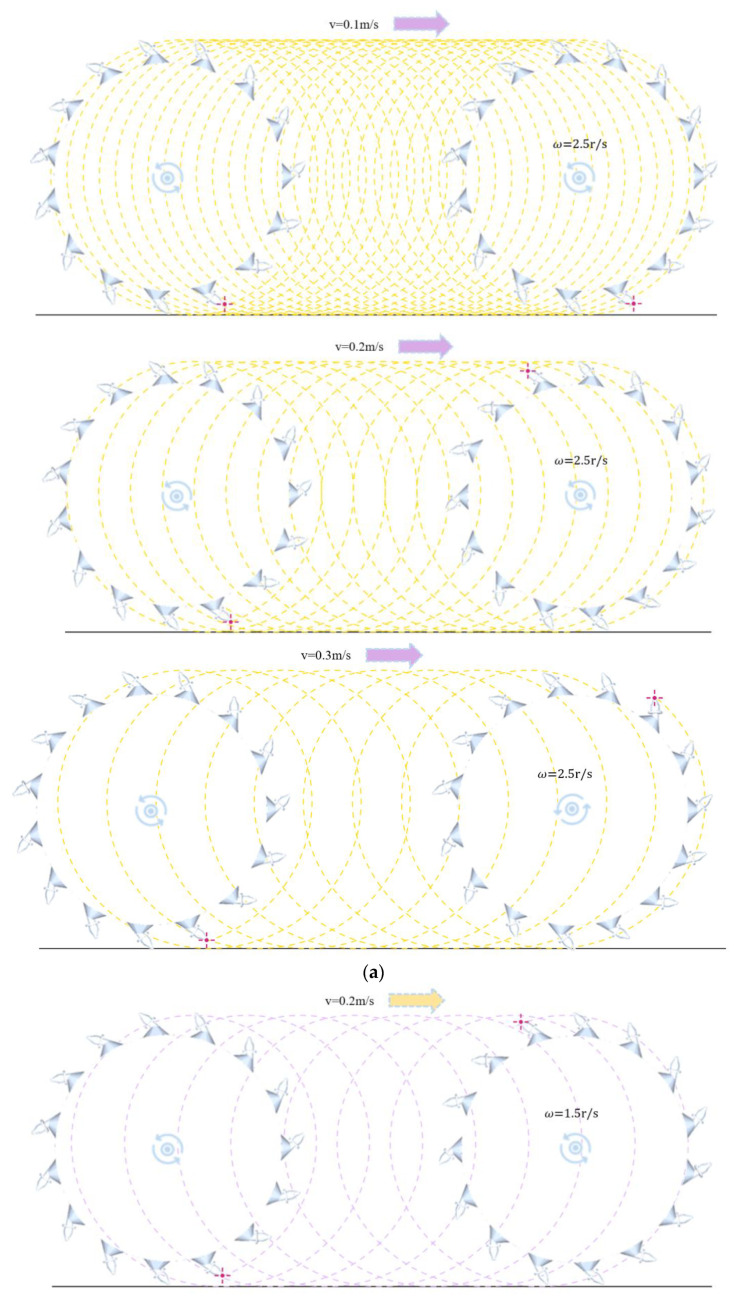

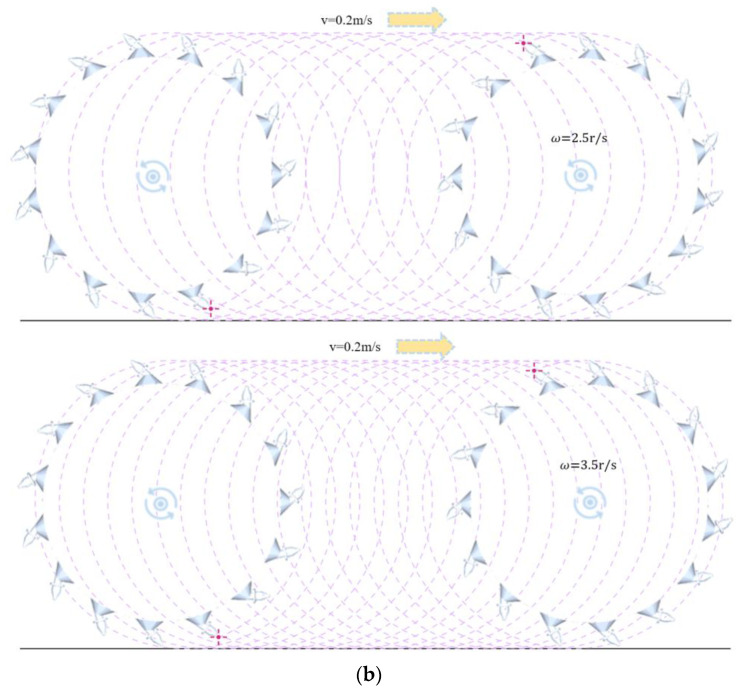
Cutter tip paths for different combinations of milling parameters: (**a**) cutter tip paths at different moving speeds (ω = 2.5 r/s); (**b**) cutter tip paths for different rotating speeds (v = 0.2 m/s).

Different cutter tip paths were generated by different combinations of milling parameters. When controlling the milling rotor moving speed and rotating speed constant, parameters can be changed to obtain each cutter tip path variation, as shown in Figure 13.

The milling path of the cutter tip is clearly indicated by the trajectory line. It is clear from Figure 13 that as the milling rotor moving speed increases, the cutter tip produces a longer milling path on the pavement in a single pass, which in turn increases the milling area. In contrast, the milling path produced by the cutter tip on the pavement in a single pass becomes shorter as the milling rotor’s rotating speed increases, resulting in a smaller milling area. Therefore, there is more RAP agglomeration as the moving speed of the milling rotor increases; longer milling paths and larger milling areas result in more resistance to the milling rotor while generating more RAP agglomeration. This is reflected in the simulation results as a consequent increase in the particle bonding ratio and force on the milling rotor. The degree of influence on the two test indexes is small as the milling rotor rotating speed increases because the amplitude of the changes in the milling path and the milling area are relatively small.

**Figure 14:**
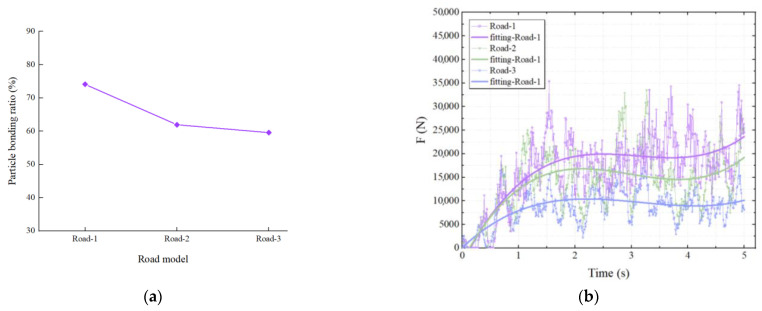
The influence of pavement service years on two test indexes: (**a**) the influence of pavement service years on the particle bonding ratio; (**b**) the influence of pavement service years on the rotor force.

The particle bonding ratio and rotor force for comparing the three pavement models at a milling moving speed of 0.2 m/s and a rotating speed of 1.5 r/s are shown in Figure 14. The particle bonding ratio and the rotor force can be found to decrease with increasing pavement service years for the same milling parameters. This is because the bonding strength of asphalt decreases with service years, resulting in a coarse aggregate bonding surface that is more susceptible to breakdown and ultimately a decrease in the particle bonding ratio. Similarly, the energy required for fracture expansion along the aggregate bonding surface during milling rotor will be reduced and it will be easier to slip and fall off, so the resistance of the rotor will be reduced.

**Figure 15:**
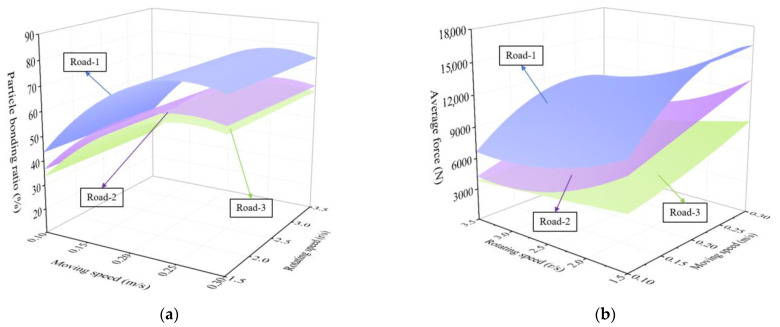
The influence of milling parameters on the particle bonding ratio and rotor average force for the three pavement model conditions: (**a**) the influence of milling parameters on the particle bonding ratio; (**b**) the influence of milling parameters on the rotor average force.

The influence of milling rotor moving speed and rotating speed on the two test indexes under the three pavement model conditions is shown in Figure 15. It can be found that the patterns of influence on the particle bonding ratio and milling rotor average force for the three pavement service year cases by milling rotor moving speed and rotating speed are overall consistent. The range of the particle bonding ratio is roughly distributed between 32% and 77%, and the range of the milling rotor average force is roughly distributed between 3800 N and 15000 N. For the overall decrease with increasing pavement service years, from Road-1 to Road-2, the overall decrease in the particle bonding ratio index is about 12% and the milling rotor average force is about 24%; from Road-2 to Road-3, the overall decrease in the particle bonding ratio index is about 3% and the milling rotor average force is about 15%. Road-1 has clearly higher test indexes than Road-2 and Road-3, and the gap between Road-2 and Road-3 is smaller, since the mechanical properties of SBS modified asphalt pavements decay relatively slowly in the later stages of service compared to the earlier stages [33].

The gap in discrete element milling simulation of SBS modified asphalt pavements with different service years has been filled by this research. The model is suitable for SBS modified asphalt pavements and needs to be validated by field tests. As this research’s results are based on simulation studies, different pavement milling projects will face the effects of differences in original pavement materials, pavement structures, pavement milling depths, and a variety of other factors, which will lead to a high degree of variability in the results [34]. Therefore, this research avoids recommending specific milling speeds, and the main objective is to elucidate the milling characteristics of SBS modified asphalt pavements with different service years. The desired milling effect to increase the recycling efficiency of RAP is to have as little RAP agglomeration as possible and to minimize aggregate crushing. That means that the particle bonding ratio must be as low as possible while at the same time ensuring that the forces on the cutter tip are as low as possible. In field milling works, as the service years of SBS modified asphalt pavement increase, it is best to control the milling moving speed at around 0.1 m/s, while appropriately increasing the rotating speed within the range of 3.1 r/s to 3.5 r/s. Based on the simulation data from this research, after validating a preliminary milling section by adjusting the milling parameters, the most suitable milling parameters are selected based on the field milling results. Selection of optimized milling parameters for the operation will have yielded higher quality RAP, which could improve the RAP recycling rate and result in economic cost savings of approximately 12.5–21.9% [35].

## 4. Conclusions

A simulation of the milling conditions for SBS modified asphalt pavement with different service years based on the discrete element method was conducted in this research. This approach reduced testing costs and improved testing efficiency. Although field milling tests could not be conducted due to objective constraints, the simulation results can provide theoretical reference for actual milling projects. Based on the simulation results, adding verification from field measurements will ultimately achieve an increase in the recycling utilization rate of SBS modified asphalt pavement recycled materials and realize sustainable green development. The main conclusions are as follows:

(1) With the constant rotating speed of the milling rotor controlled, the single milling path of the cutter tip becomes longer as the milling rotor moving speed increases, thereby increasing the milling area. Conversely, with a fixed milling rotor moving speed, as the rotating speed of the milling rotor increases, the cutter tip milling path becomes shorter, resulting in a reduced milling area.
(2) As the moving speed of the milling rotor increased from 0.1 m/s to 0.3 m/s, longer milling paths and larger milling areas result in more RAP agglomeration along with greater resistance to the milling rotor. This is reflected in the simulation results by an increase of about 30% for the particle bonding rate and an increase of about 8388 N for the rotor average force. The degree of influence on the two test indexes is small as the milling rotor rotating speed increased from 1.5 r/s to 3.5 r/s because the amplitude of the changes in the milling path and the milling area is relatively small. The particle bonding ratio decreased by about 2.38% and the milling rotor average force decreased by about 4304 N.
(3) The overall pattern of influence exerted by the milling rotor moving speed and rotating speed on the particle bonding ratio and rotor average force applied to the milling rotor remained consistent as the service years of the pavement increased. From 2~3 years pavements to 7~8 years pavements, the overall reduction in the particle bonding ratio index is about 12% and the milling rotor average force is about 24%; from 7~8 years pavements to 11~12 years pavements, the overall reduction in the particle bonding ratio index is about 3% and the milling rotor average force is about 15%.

## Figures and Tables

**Table 1:** Technical indicators for SBS modified asphalt.

Item	Unit	Test Value	Specification	Test Method
Penetration (25 °C, 100 g, 5 s)	0.1 mm	70.8	60~80	T 0604
Softening point	°C	58.6	≥55	T 0606
Residual ductility at 5 °C	cm	42.1	≥30	T 0605

**Table 2:** Three major indexes of SBS modified asphalt with different RTFOT aging times.

RTFOT Aging Time (min)	Softening Point (°C)	Penetration (25 °C, 100 g, 5 s)	Residual Ductility at 5 °C (cm)
180	61.3	41.1	22.9
360	72.5	29.2	7.1
600	77.8	26.8	5.8

**Table 3:** Technical indicators of coarse aggregates, fine aggregates, and mineral powders.

Material Type	Test Item		Unit	Standard Requirement	Test Result			Assess	Testing Method
Coarse Aggregate	Specifications		mm		15~25	5~15	3~5		
	Apparent Relative Density		g/cm^3^	≥2.60	2.867	2.718	2.825	Qualified	T 0304
	Bulk Relative Density		g/cm^3^	Measured	2.818	2.648	2.732	Qualified	T 0304
	Water Absorption Rate		%	≤2.0	0.62	0.97	1.2	Qualified	T 0304
	Adhesion to Asphalt		Stage	≥5	5			Qualified	T 0616
	Crushing Value		%	≤28	16.2			Qualified	T 0316
	Elongated and Flaky Particle Content		%	≤18	8.6			Qualified	T 0312
	Soft Stone Content		%	≤3	2.3			Qualified	T 0320
	Sturdiness		%	≤12	7			Qualified	T 0314
Fine Aggregate	Apparent Density		g/cm^3^	Measured	2.701			Qualified	T 0328
	Apparent Relative Density		—	≥2.50	2.705			—	T 0328
	Robustness (>0.3 mm)		%	≤12	8.8			Qualified	T 0340
	Sand Equivalent		%	≥60	63			Qualified	T 0334
Mineral Powder	Apparent Density		g/cm^3^	Measured	2.698			Qualified	T 0352
	Water Content		%	≤1	0.33			Qualified	T 0103 Drying Method
	Particle Size Range	<0.6 mm	%	100	100			Qualified	T 0351
		<0.15 mm	%	90~100	96.6			Qualified	
		<0.075 mm	%	75~100	85.8			Qualified	
	Appearance		—	Free from Agglomeration and Caking	Free from Agglomeration and Caking			Qualified	—
	Hydrophilic Coefficient		—	<1.0	0.8			Qualified	T 0353

**Table 4:** Splitting tensile strength of Marshall specimens.

RTFOT Aging Time (min)	Splitting Tensile Strength (*R_T_*/MPa)				Standard Error
	1	2	3	Average	
180	0.758	0.766	0.763	0.762	0.0040
360	0.527	0.536	0.530	0.531	0.0046
600	0.459	0.483	0.474	0.472	0.0120

**Table 5:** The values of the bonding parameters.

Average Splitting Tensile Strength (*R_T_*/MPa)	$X_{1}$ /(N∙m^−3^)	$X_{2}$ /(N∙m^−3^)	$X_{3}$ /Pa	$X_{4}$ /Pa
0.762	1.31 × 10^10^	2.16 × 10^10^	4.12 × 10^9^	1.94 × 10^9^
0.531	1.31 × 10^10^	1.27 × 10^10^	2.83 × 10^9^	5.53 × 10^9^
0.472	1.31 × 10^10^	1.39 × 10^10^	2.58 × 10^9^	1.25 × 10^9^

**Table 6:** Experimental program for milling characterization research of old asphalt pavements.

Test Number	Pavement Model	Test Factors	
		Moving Speed v (m/s)	Rotating Speed ω (r/s)
1	Road-1	0.1	1.5
2	Road-1	0.1	2.5
3	Road-1	0.1	3.5
4	Road-1	0.2	1.5
5	Road-1	0.2	2.5
6	Road-1	0.2	3.5
7	Road-1	0.3	1.5
8	Road-1	0.3	2.5
9	Road-1	0.3	3.5
10	Road-2	0.1	1.5
11	Road-2	0.1	2.5
12	Road-2	0.1	3.5
13	Road-2	0.2	1.5
14	Road-2	0.2	2.5
15	Road-2	0.2	3.5
16	Road-2	0.3	1.5
17	Road-2	0.3	2.5
18	Road-2	0.3	3.5
19	Road-3	0.1	1.5
20	Road-3	0.1	2.5
21	Road-3	0.1	3.5
22	Road-3	0.2	1.5
23	Road-3	0.2	2.5
24	Road-3	0.2	3.5
25	Road-3	0.3	1.5
26	Road-3	0.3	2.5
27	Road-3	0.3	3.5

## Data Availability

The original contributions presented in this study are included in the article. Further inquiries can be directed to the corresponding author.
